# Photodynamic Therapy for the Treatment of Glioblastoma

**DOI:** 10.3389/fsurg.2019.00081

**Published:** 2020-01-21

**Authors:** Samuel W. Cramer, Clark C. Chen

**Affiliations:** Department of Neurosurgery, University of Minnesota, Minneapolis, MN, United States

**Keywords:** brain tumor, photodynamic therapy (PDT), glioblastoma multiforme (GBM), tumor-targeting, neurosurgery

## Abstract

Glioblastoma is the most common form of adult brain cancer and remains one of the deadliest of human cancers. The current standard-of-care involves maximal tumor resection followed by treatment with concurrent radiation therapy and the chemotherapy temozolomide. Recurrence after this therapy is nearly universal within 2 years of diagnosis. Notably, >80% of recurrence is found in the region adjacent to the resection cavity. The need for improved local control in this region, thus remains unmet. The FDA approval of 5-aminolevulinic acid (5-ALA) for fluorescence guided glioblastoma resection renewed interests in leveraging this agent as a means to administer photodynamic therapy (PDT). Here we review the general principles of PDT as well as the available literature on PDT as a glioblastoma therapeutic platform.

## Introduction

Glioblastoma is a malignant central nervous system (CNS) neoplasm with histologic features resembling astrocytic cells. It is the most common form of primary brain cancer, with an incidence of 3.19 per 100,000 people in the United States (1). Glioblastomas are aggressive and infiltrative, with microscopic extension into normal brain parenchyma (2, 3). Invading tumor cells exhibit characteristic migratory patterns, including spread beneath the pial margin (subpial spread), along neurons (perineuronal spread), along cerebrovasculature (perivascular satellitosis), or along white matter tracts (intrafascicular spread) (4). Microscopic, infiltrating cells are found centimeters from the margin of the visible tumor mass (5). As such, surgical resection is not curative. The current standard-of-care involves maximal, safe surgical resection followed by concurrent chemotherapy (temozolomide) and fractionated radiotherapy (FRT) (6–9). The overall prognosis for glioblastoma patients is poor, with reported median survival of 14.6 months (7), and tumor recurrence near universal.

The observation that >80.0% of the recurrences are located adjacent to the resection cavity suggests utility for therapeutic platforms targeting this region (10–13). The recent United States Food and Drug Administration (FDA) approval of 5-aminolevulinic acid (5-ALA) for fluorescence guided resection (FGR) of tumors renewed interests in leveraging this agent as a means to administer photodynamic therapy (PDT). In principle PDT to the resection cavity can minimize the risk of local recurrence. In this article, we will review the current state of the literature as it pertains to PDT as a glioblastoma therapeutic platform.

## Methods

The goal of this article is to provide a current state of the art review of photodynamic therapies for the treatment of glioblastoma. To this end, we aim to provide an overview of the development of photodynamic therapy for glioblastoma, describe the physical mechanism of the therapeutic approach, describe known interactions between PDT and pharmacological treatments, as well as predict future developments in this field. Therefore, a comprehensive literature search was performed in PubMed (MEDLINE) using MeSH terms “photodynamic therapy gliomas” which resulted in 480 articles. The type of publications considered included clinical and pre-clinical trials, systematic reviews and case series. Relevant publications were then selected based upon validated academic metrics including journal impact factor and i10 factor.

### Principle of Photodynamic Therapy (PDT)

**Figure 1:**
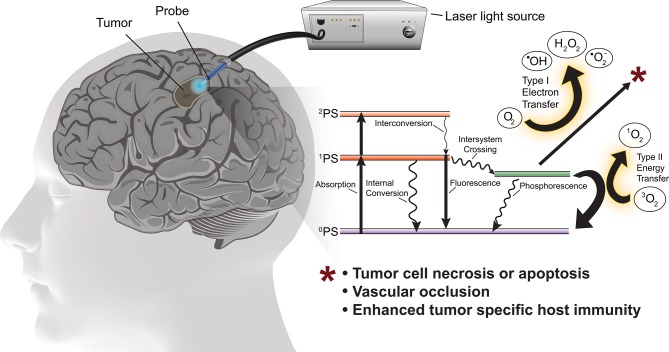
Schematic of PDT for the treatment of glioblastoma with simplified energy diagram of the oxygen dependent photodynamic response. The photosensitizer in the ground state (^0^PS) is excited to one of two states by the appropriate wavelength and power photostimulation, the first excited singlet state (^1^PS) or the second excited singlet state (^2^PS). The ^1^PS may then convert to the excited triplet state (^3^PS) via intersystem crossing. In the presence of molecular oxygen, the ^3^PS may undergo Type I or Type II redox reactions producing reactive oxygen species which cause tumor cell death.

PDT involves photo-activation of a photosensitizer molecule that is selectively incorporated into neoplastic cells. Photo-irradiation activates the photosensitizer by transfer of energy to the sensitizer resulting in excitation of molecular oxygen to a singlet or triplet state. In the singlet state the energy is converted to heat (internal conversion) or is emitted as light (fluorescence). In the triplet state, the energy generates reactive oxygen species (ROS) necessary to induce cell death (Figure 1). ROS rapidly react with macromolecules containing unsaturated double bonds, including proteins, unsaturated fatty acids and cholesterols. These reactions damage the membranes of intracellular organelles, such as mitochondria, lysosomes, and the endoplasmic reticulum (8), ultimately triggering necrosis, apoptosis, local ischemia (due to occlusion of neoplastic vessels) as well as subsequent immunological reactions (14).

Singlet oxygen diffuses over short distances (i.e., ~0.02–1.00 μm) and has a limited lifespan (i.e., ~0.04–4.0 μs) contributing to local tumor ablation while minimizing risk of damage to adjacent normal tissue (15). The type of photosensitizer and photo-activation determines the specific intracellular components affected as well as the degree and type of damage incurred by those components.

Like ionizing radiation, the cytotoxic effects of PDT requires the presence of molecular oxygen. Thus, the degree of oxygenation with the tumor microenvironment is a key determinant of PDT's tumoricidal activity. In this context, PDT is often delivered through multi-session treatment in order to facilitate re-oxygenation between treatments (16, 17). Pre-clinical and clinical trials of PDT performed in combination with hyperbaric oxygen demonstrate improved tumoricidal activity (18).

Though both radiation and PDT require molecular oxygen for their respective anti-neoplastic activities, their modes of action fundamentally differ. The available data suggest that ionizing radiation triggers cell death through induction of DNA damage (19). In contrast, the predominant model of cytotoxicity by PDT involves damage of cell membranes, proteins, and organelles (20). As such, PDT potentially synergizes with DNA damaging agents routinely used as standard-of-care treatment for glioblastoma (21).

### Photo-Activation

The light source may be incoherent or coherent (i.e., laser). The efficacy of PDT is not affected by the coherence of the light source. The emission wavelength of the light source is adjusted to the absorption spectrum of the photosensitizer. Photo-irradiation with longer wavelength light is preferred because it penetrates more deeply and delivers sufficiently energetic photons to activate the photosensitizer. Given the excitation peaks of clinically available photosensitizers and the limitations of photon propagation through biological tissues, PDT generally utilizes wavelengths between ~400 and 900 nm, with an optimal window of 600–800 nm (15). PDT stimulation may be applied in a continuous or pulsed fashion (14), with consideration that pulsed delivery may facilitate tumor re-oxygenation between pulses.

### First, Second, and Third Generation Photosensitizers

First-generation photosensitizer molecules consist of naturally occurring porphyrins, including hematoporphyrin (Table 1). These compounds have a strong absorption around 400 nm but have limited excitation absorption at longer wavelengths of light (22). HpD is an example of a first generation photosensitizer. It consists of a proprietary combination of monomers, dimers and oligomers derived from hematoporphyrin (23). HpD is an inefficient producer of singlet oxygen requiring extended photo-stimulation to achieve adequate therapeutic effect (23).

**Table 1:** Properties of clinically relevant photosensitizers.

**Photosensitizer**	**Trade name(s)**	**Excitation wavelength(s)**	**Treatment window^a^**	**Clearance time**	**Side effects**
Talaporfin sodium	Laserphyrin, Aptocine™, Litx™, LS11, Photolon®	664	2–4 h	15 days	Skin sensitization for 2 weeks
HpD	Photofrin®, Photogem®	408, 510, 630^b^	24–48 h	4–6 weeks	Skin sensitization for several weeks
5-ALA (PpIX)	Levulan®	410, 510, 635^b^	4–8 h	2 days	Skin sensitization for few days, nausea, elevated liver enzymes, anemia,
Porfimer sodium	Photofrin II®	630	48–150 h	4–6 weeks	Skin sensitization for several weeks
BOPP	n/a	630	24 h	4–6 weeks	Skin sensitization for several weeks, thrombopenia
Temoporfin	Foscan®	652	4 days	2–6 weeks	Skin sensitization for several weeks

a *Latency after drug administration and accumulation of photosensitizer in the tumor*.

b *Optimal excitation wavelength for clinical application*.

*HpD, Hematoporphyrin derivative; BOPP, Boronated porphyrin*.

Second-generation photosensitizers were developed to overcome the inherent limitations of first-generation sensitizers. They are usually activated by wavelengths >600 nm and are more potent in generating singlet oxygen. Chlorins (talaporfin sodium and temoporfin) and 5-Aminolevulinic acid (5-ALA) are examples of second-generation photosensitizers (15).

Talaporfin sodium and temoporfin are clinically used to treat dermatologic diseases. Talaporfin is water soluble and administered intravenously. As such, it is quickly cleared from the body. Talaporfin is activated by 664 nm light (23, 24). Temoporfin is the most potent of the clinically available photosensitizers (activated by 652 nm photostimulation). Temoporfin is generally well-tolerated but does confer photosensitivity for up to 6 weeks post-administration.

Among the commercially available photosensitizers, 5-ALA is commonly utilized (25). The clinical utility is driven by oral bioavailability and a highly favorable safety profile. Pertaining to glioblastomas, 5-ALA exhibits high selectivity in terms of preferential accumulation in malignant gliomas (15). 5-ALA is the first compound in the synthesis of porphyrin, a component required for heme synthesis. Porphyrins are assembled into porphobilinogen, which is converted to protoporphyrin IX (PpIX) by porphobilinogen deaminase. The expression of this deaminase is elevated in glioblastomas, resulting in increased synthesis of PpIX. PpIX is normally converted to heme by the enzyme ferrochelatase (26). Decreased expression of ferrochelatase in glioblastoma arrests this conversion, further augmenting the accumulation of PpIX in glioblastomas (15).

PpIX absorbs blue light (404 nm) and emits fluorescence in the red spectrum (635 nm). When excited by 635 nm light, PpIX generates triplet oxygen and produces cytotoxicity as a photosensitizer (27). Of note, there is significant inter-tumoral heterogeneity in PpIX concentration after 5-ALA administration (25). The molecular underpinning of this variation remains poorly understood though may involve inter-tumoral expression differences in ATP-binding cassette transporters (28).

Third generation photosensitizers are characterized by enhanced tumor cell selectivity achieved through the conjugation of modifiers including nanoparticles and antibodies (15, 23). Development of third generation photosensitizers has also emphasized the design of prodrugs that are only activated by neoplastic cells. The goal of rational design of third generation photosensitizers is to reduce off target effects while optimizing pharmacokinetics and excitation-absorption properties to maximize the effective PDT window while minimizing side-effects. At this time, no third generation photosensitizers are approved for PDT in humans.

### Blood-Brain Barrier

In the normal brain, endothelial cells exhibit morphological specializations including the expression of tight junctions that contribute to the formation of the blood-brain barrier (BBB). There is no active transport system for 5-ALA across the BBB. As such, there is little spontaneous diffusion of 5-ALA into normal brain tissue (25). Break-down in the BBB which frequently occurs in the glioblastoma microenvironment facilitates diffusion of 5-ALA into the tumor mass. In this context, 5-ALA guided surgical resection facilitates removal of the contrast enhancement, which is typically observed in these regions of BBB breakdown.

Whether 5-ALA is a true proxy for tumor mass or simply the region of BBB breakdown remains an open question. The therapeutic window for 5-ALA mediated PDT depends on the extent that PpIX is preferentially accumulated in the glioblastoma cell relative to the cellular constituents of the tumor microenvironment (29).

### Medications That May Affect PDT for Glioblastoma

Glioblastoma patients are commonly prescribed anti-epileptic drugs (AEDs, such as phenytoin and levetiracetam) as well as corticosteroid therapy. There are pre-clinical data that suggests these drugs interact with 5-ALA metabolism. In pre-clinical investigation, phenytoin administration after 5-ALA infusion suppressed PpIX synthesis by 31.0% (30). In contrast, levetiracetam did not affect PpIX synthesis or the response to PDT (30).

The interaction between corticosteroid and PpIX synthesis is more complex. PpIX production in response to 5-ALA was reduced by dexamethasone administration. However, the cellular retention of PpIX retention was increased (30). The complexity is further layered in the observation that corticosteroid administration reduced BBB permeability, which may hinder uptake of 5-ALA (15). Pre-clinical studies suggest that this effect is most prominent for dexamethasone (a commonly used corticosteroid in glioblastoma patients) with doses exceeding 12 mg per day (31).

Additionally, other FDA approved medications increase PpIX accumulation in tumor cells, including iron chelators (deferoxamine and deferiprone), vitamin D, ciprofloxacin, 5-fluorouracil, and febuxostat. Combination of these drugs has been proposed as a means to augment efficacy of 5-ALA in PDT (32).

## Clinical Applications

Infiltrating glioblastoma cells can be found 4.0 cm beyond the border of radiologically or histologically identifiable tumor lesions (33). Infiltrating tumor growth and extent of resection is difficult to assess intraoperatively and infiltrative tumor cells are always left behind when using traditional surgical and imaging approaches. Therefore, a method that affords the visual identification of neoplastic tissue and the simultaneous ability to selectively destroy that tissue would likely improve the success of glioblastoma resection. The joint clinical application of fluorescence guided surgery (FGS) and PDT confers the ability to both visualize tumor cells and selectively destroy them.

### Interstitial PDT

Interstitial PDT (iPDT) is applied via the stereotactic insertion of fiber optic cable(s) into the tumor to deliver photostimulation to the tumor mass after the patient has been administered a photosensitizer (25). The application of iPDT is similar to laser interstitial thermal therapy (LITT) for the treatment of glioblastoma as both are minimally invasive stereotactic techniques, however, iPDT has the added benefit of selective neoplastic cell targeting. Several technical considerations are unique to iPDT. For example, selecting a light diffuser with the appropriate geometry to apply optimal photostimulation to the target tumor, determining the optimal number of diffusers to insert into the tumor to maximize therapy while minimizing the harm associated with insertion of the diffuser through normal brain tissue and, finally, proper selection of tumors of the appropriate size, anatomic location, and geometry to maximize the safety and efficacy of iPDT.

A challenge of iPDT is the even delivery of photostimulation to achieve adequate fluence over a maximal volume of tumor without causing thermal injury to the normal brain tissue. Modeling experiments have examined light delivery and determined the optimal geometry of light guides for the delivery of iPDT. Cylindrical light diffusers have a larger emitting surface area with a lower fluence rate at the tissue/light emitter interface than flat cleaved fibers (34). Therefore, light delivery via cylindrical diffuser improves photon distribution with a reduced sensitivity to local tissue absorption variability thereby distributing photostimulation over a greater tissue volume than flat, cleaved fibers. However, the light fluence drops off more rapidly from the flat fiber which is useful when treating a tumor in close proximity to eloquent brain tissue. Therefore, the geometry of the light diffuser as well as the total number of diffusers needed to safely treat a tumor are factors to consider preoperatively in order to achieve optimal iPDT.

The dose of light delivered during PDT is another important consideration. A dosimetry model was developed which is specific to 5-ALA but the underlying principle must be considered for iPDT performed with any photosensitizer. To achieve the maximal therapeutic effect of PDT, the goal is to achieve “advanced photobleaching” (based on an established dosimetry model of the same name) of the photosensitizer. Advanced photobleaching is defined as the fluence rate at which causes ≥95% photobleaching of photosensitizer and is associated with better outcomes (35, 36). In the case of 5-ALA mediated PDT, simulations suggest advanced photobleaching is achieved to a distance of ~4 mm from the surface of a light diffuser emitting a power of 200 mW/cm for 1 h. Based upon the estimated volume of tissue affected by photo-irradiation, the optimal interfiber distance of the photo diffusers for iPDT is ~10 mm and maximum power of photostimulation should not exceed 200 mW/cm as the threshold at which the risk of increasing tissue temperatures >48°C (the threshold at which thermal side-effects become a factor) increases significantly (25, 37).

Software for optimization of iPDT delivery has been in development for several decades (31, 36). One approach utilizes the co-registration of contrasted magnetic resonance imaging and positron emission tomography imaging with stereotactic computed tomography images to allow virtual trajectory planning and positioning of light diffusers within tumors (37). The goal is to virtually plan the implantation of the optimal number of light diffusers for tumor ablation without causing injury to the adjacent vasculature or traversing eloquent cortex.

### Post-resection PDT

After maximal safe tumor resection, PDT may be applied to the resection cavity in the operating room or during post-operative recovery. Cavitary PDT is commonly applied by placing a balloon filled with diffusing liquid (typically a lipid suspension) coupled to a fiber optic guide and an external light source into the intracranial resection cavity. After tumor resection, the balloon is positioned in the cavity and inflated to conform with the geometry of the cavity without causing excessive compression of surrounding brain tissue. In one photostimulation paradigm utilizing the diffuser balloon, total treatment time was derived from the volume of the diffusing media in the balloon and applied in five fractions to the tumor. Between photo-irradiation fractions, 2.0 min pauses are applied allowing brain tissue reoxygenation. All photostimulation fractions are delivered in the operating room. This is the approach being utilized in the Intraoperative Photodynamic Therapy of glioblastoma (INDYGO) clinical trial which is currently ongoing (17).

Another method for cavitary PDT is to apply fractions of photostimulation out of the operating room during the post-operative recovery period. After FGS, a balloon diffuser is placed in the resection cavity and inflated with a radio-opaque lipid emulsion until the resection cavity is filled. Fluoroscopy is used to verify balloon inflation and later complete deflation, prior to removal. The first PDT treatment is applied in the recovery area with daily PDT treatments delivered subsequently at the bedside for a total of 5 treatments. A key consideration for applying PDT over a prolonged time period is the effective half-life of the photosensitizer (in this example porfimer sodium was used). After the total number of treatments are delivered, the balloon diffuser is deflated and removed at the bedside (38, 39).

## Clinical Outcomes Of PDT Treated Glioblastomas

Outcomes after PDT in glioblastoma patients are generally favorable compared to historical data, however, the quality of the studies is limited by the lack of randomized controlled trials. The Royal Melbourne Hospital group has the most extensive clinical experience with PDT for gliomas with a series of more than 350 patients and report overall survival rates of those with newly diagnosed and recurrent glioblastomas of 28.0 and 40.0%, at 2 years and 22.0 and 34.0% at 5 years, respectively, an improvement compared to historical controls. Similarly, a meta-analysis of more than 1,000 patients enrolled in observational studies of PDT for high-grade gliomas reported median survival of newly diagnosed and recurrent glioblastomas of 16.1 and 10.3 months, respectively (40). A summary of a summary of clinical trials evaluating PDT for the treatment of glioblastomas is found in Table 2.

**Table 2:** Summary of clinical trials using PDT for the treatment of GBM.

**References**	**Number of patients**		**Photosensitizer**		**Photo-irradiation**		**Median overall survival (mo)**
	**New GBM**	**rGBM**	**Drug**	**Dose, route of administration**	**Wavelength, nm**	**Energy density (J/cm^**2**^)**	
Stupp et al. (6)	287		n/a	n/a	n/a	n/a	14.6^c^ newly diagnosed
Akimoto et al. (41)	6	8	Talaporfin sodium	40 mg/m^2^, IV	664	27	n/a
Beck et al. (37)^a^		10	5-ALA	20 mg/kg, PO	633	100	15 rGBM
Eljamel et al. (39)	13		5-ALA and Porfimer sodium	2 mg/kg Photofrin IV; 20 mg/kg 5-ALA, PO	630	100	13.2^d^ newly diagnosed
Johansson et al. (36)^a^	1	4	5-ALA	20–30 mg/kg	635	720 J/cm	n/a
Kaye et al. (42)	13	6	HpD	5 mg/kg, IV	630	70–120 or 120–230 J/cm^2^	n/a
Kostron et al. (43)	18		HpD	1 mg/cm^3^ of tumor IA and/or 1 mg/cm^3^ into tumor and/or IV mg/cm^3^	630	40–120	n/a
Kostron et al. (44)		26	Temoporfin	0.15 mg/kg, IV	652	20	8.5 rGBM
McCulloch et al. (45)	9		HpD	5 mg/kg, IV	627.8^b^	n/a	n/a
Muller and Wilson (46)	17		HpD or Porfimer sodium	1.4–2.7 mg/kg, IV	630	8–68	n/a
Muller and Wilson (47)		32	HpD or Porfimer sodium	5 mg/kg, IV; 2 mg/kg, IV	630	8–110	7.5 (29.5 from diagnosis) rGBM
Muller and Wilson (48)	12	37	Porfimer sodium	2 mg/kg, IV	n/a	58 (mean)	8.25 newly diagnosed, 7.25 rGBM
Muller and Wilson (49)	11		Porfimer sodium	2 mg/kg, IV	630	8–110	9.25 newly diagnosed
Muller et al. (50)		37	Porfimer sodium	2 mg/kg, IV	n/a	8–150	7.75 rGBM
Muragaki et al. (51)	13		Talaporfin sodium	40 mg/m^2^, IV	664	27	24.8 newly diagnosed
Nitta et al. (52)	30		Talaporfin sodium	40 mg/m^2^, IV	664	27	27.4 newly diagnosed
Origitano et al. (31)^a^		8	Porfimer sodium	2 mg/kg, IV	630	50 (100 J/cm interstitial)	n/a
Popovic et al. (53)	38	40	HpD	5 mg/kg, IV	628	72–260	24 newly diagnosed, 9 rGBM
Powers et al. (54)	2		Porfimer sodium	2 mg/kg, IV	630	400 J/cm	n/a
Rosenthal et al. (55)	7	9	Boronated porphyrin	0.25–8.0 mg/kg, IV	630	25–100	5 newly diagnosed, 11 rGBM
Schwartz et al. (56)^a^	15		5-ALA	20–30 mg/kg, PO	633	12.960 J	n/a
Stylli et al. (57)	58		HpD	5 mg/kg, IV	n/a^b^	240 J/cm^2^ (median)	24 newly diagnosed
Stylli et al. (58)	31	55	HpD	5 mg/kg, IV	n/a^b^	230 J/cm^2^ (median)	14.3 newly diagnosed, 14.9 rGBM
Vanaclocha et al. (59)	20		Porfimer sodium or temoporfin	2 mg/kg, IV; 0.15 mg/kg, IV	630; 652	20–75	17 newly diagnosed

a *iPDT*.

b *Multiple lasers used*.

c *Reference survival time for newly diagnosed GBM*.

d *Mean*.

*rGBM, recurrent GBM; HpD, hematoporphyrin derivative*.

### HpD Mediated PDT for Glioblastoma

An early study evaluating the efficacy of HpD mediated PDT enrolled 18 glioblastoma patients. HpD was administered via direct arterial puncture during preoperative angiogram, IV or directly into the tumor during craniotomy for tumor resection. Cavitary PDT was applied intraoperatively after tumor resection. Patients were brought back to the operating room 3 days later for redo-craniotomy and administered another round of PDT. At publication, six patients with primary glioblastomas were surviving at 22.0 months (43).

The effects of HpD or porfimer sodium mediated PDT on glioblastomas was evaluated in 17 patients. Patients were administered the photosensitizer 18–24 h prior to undergoing maximal tumor resection and intraoperative, cavitary PDT was applied via an inflatable balloon diffuser. For glioblastoma patients that died during follow-up, mean survival was 6.3 months post-PDT (46).

HpD concentration in tumor tissue compared to survival after PDT was evaluated in 58 glioblastoma patients. Patients underwent maximal safe tumor resection, then intraoperative, cavitary PDT was administered by filling the resection cavity with a continuously circulating lipid emulsion while photostimulation was applied. There was a strong association between HpD uptake and survival among treated patients (Hazard Ratio = 0.26, *p* = 0.001) and the median overall survival for glioblastoma patients after PDT was 24.0 months (57). A similar study evaluating HpD mediated PDT for high grade gliomas including 78 glioblastoma patients was performed. Patients underwent maximal safe tumor resection followed by intra-operative photoirradiation. The median overall survival for glioblastoma patients treated with PDT was 14.3 months (58).

### Porfimer Sodium Mediated PDT for Glioblastoma

A case series reports the efficacy of porfimer sodium mediated PDT for newly diagnosed and recurrent glioblastomas in 49 patients. After the maximal tumor resection, either a balloon diffuser was placed in the resection cavity or the resection cavity itself was filled with a continuous infusion of lipid emulsion and photo-irradiation was applied. The median survival of glioblastoma patients was 30 weeks, with 1- and 2-years actuarial survival of 22.0 and 2.0%, respectively (48).

A small study was conducted to evaluate porfimer sodium or temoporfin mediated PDT for malignant primary brain tumors, including 20 glioblastoma patients. Patients underwent tumor resection followed by photo-irradiation to the resection cavity. Light was delivered with a 630 nm laser with a fluence of 75 J/cm^2^ in porfimer sodium patients and at 652 nm with a fluence of 20 J/cm^2^ in the temoporfin patients. A continuous infusion of lipid emulsion was applied to the resection cavity during photo-irradiation to minimize the risk of thermal injury to brain tissue. Post-operatively, patients were kept in a unique ICU room with no exposure to sun-light. Sunlight was avoided for 4 weeks (porfimer sodium) or 2 weeks (temoporfin). Patients received standard temozolomide chemotherapy and FRT post-operatively. Median OS for these patients was 17 months (59).

A phase II study evaluated porfimer sodium mediated PDT for newly diagnosed and recurrent supratentorial gliomas including 37 recurrent and 11 newly diagnosed glioblastomas. Subjects enrolled in the study underwent tumor resection followed by intraoperative placement of expandable balloon irradiator filled with a light dispersion medium for photo-irradiation (49, 50). Those with recurrent glioblastomas who had failed prior surgical resection and FRT (with or without chemotherapy) underwent PDT while patients with newly diagnosed tumors underwent surgical resection with intraoperative PDT. The median survival for newly diagnosed glioblastoma patients was 7.75 months while those with recurrent glioblastomas had a median survival of 9.25 months.

The application of PDT as treatment for glioblastomas was first evaluated in a randomized, controlled trial by Muller and Willson (40, 48). The treatment arm enrolled 43 patients who underwent glioblastoma resection followed by porfimer sodium mediated PDT and was compared to 34 patients who underwent tumor resection alone. Post-operative FRT was administered to all patients. Median survival was 11.0 months (95% CI 6.0–14.0 months) in the treatment group compared to 8.0 months (95% CI 3.0–10.0 months) in the control group. A 38.0% increase in median survival with PDT as well as >6.0-months survival rate in the treatment group were statistically significant, but Kaplan–Meier curves crossed over at 15 months (14, 40, 48).

Eljamel et al. conducted a single-center, randomized controlled phase III trial to evaluate porfimer sodium mediated PDT after 5-ALA FGS for newly diagnosed glioblastomas. In the study, 13 patients underwent FGS for glioblastoma resection followed by intracavitary placement of a balloon diffuser to provide repetitive PDT (1 session per day, 100 J/cm^2^ applied per session) for 5 days during the post-operative period. The control arm underwent FGS for tumor resection without PDT. Post-operatively, all patients underwent FRT and were followed clinically and radiographically every 3 months until death. There was no statistically significant difference in the frequency of adjuvant and salvage treatments between the two cohorts. The mean survival of patients in the PDT and surgery only groups was 52.8 weeks (95% CI 40.0–65.0 weeks) and 24.2 weeks (95% CI 18.0–30.0 weeks), respectively (*p* < 0.001). Despite an overall worse functional status in the study group prior to FGS and PDT, their functional status improved to a much higher level post-operatively compared to the surgery only group. There was no residual tumor on discharge in 10 out of 13 patients in the PDT group and in 4 out of 14 patients in the surgery only group. There was also no difference between the groups in the average length of stay in the hospital or in the complication rate.

### Talaporfin Sodium Mediated PDT for Glioblastoma

Talaporfin sodium accumulates selectively in high grade gliomas and is useful for intraoperative photodiagnosis of malignant brain tumors (60). Furthermore, several small case series report the safety and efficacy of talaporfin sodium mediated PDT as glioblastoma treatment. Thirty newly diagnosed glioblastoma patients treated with PDT in addition to standard FRT and temozolomide were compared to 164 patients with newly diagnosed glioblastomas who received standard therapy alone. The median survival time was 27.4 months for PDT patients compared to 22.1 months for those receiving standard therapy (52).

Another case series reported a median survival of 26.0 months (with one patient surviving >38.0 months) for four newly diagnosed glioblastoma patients treated with maximal safe tumor resection followed by talaporfin sodium mediated PDT while six patients with recurrent glioblastomas who underwent the same treatment had a median survival of 8.5 months (41). Muragaki et al. report their experience with talaporfin sodium PDT for the treatment of newly diagnosed or recurrent malignant primary brain tumors (including 13 glioblastoma patients). Patients underwent craniotomy and tumor resection followed by intraoperative cavitary PDT. The photostimulation was targeted to regions with high risk of tumor recurrence including the genu of the corpus callosum. In total, 1–3 regions within the resection cavity were targeted with photo-irradiated. All patients with newly diagnosed glioblastomas underwent FRT and adjuvant chemotherapy with temozolomide in addition to PDT. The median overall survival for patients with newly diagnosed glioblastomas was of 24.8 months with a median progression free survival of 12.0 months (51).

### 5-ALA Mediated PDT for Glioblastoma

Two series report their experience with 5-ALA mediated iPDT for malignant gliomas. In a pilot study, the efficacy of 5-ALA mediated iPDT for small (maximum diameter <3 cm), circumscribed recurrent malignant gliomas was evaluated in 10 patients. Based upon 3-D photoirradiation simulations during preoperative planning, 4–6 fiber diffusers were stereotactically placed per patient to achieve complete photo-irradiation of the tumors. The 1-year survival rate was 60.0% with a median survival of 15.0 months (37). A similar series in 15 patients with small newly diagnosed (<4 cm) and unresectable glioblastomas who underwent 5-ALA iPDT and were compared to glioblastoma patients (*n* = 112) who underwent complete tumor resection alone. All patients received standard radiotherapy and temozolomide. The iPDT group demonstrated significantly longer median progression free survival of 16.0 vs. 10.2 months and a 3-years survival of 56.0 vs. 21.0% (56). Of note, 6 of the 15 patients in the iPDT group experienced progression free survival >30 months.

### Boronated Porphyrin and Temoporfin Mediated PDT for Glioblastoma

A phase I trial evaluated the safety of boronated porphyrin (BOPP) for the treatment of high-grade gliomas including seven patients with newly diagnosed and 9 patients with recurrent glioblastomas. The dose of BOPP and photo-irradiation were varied incrementally with the goal of determining the maximum safe doses. The median overall survival for newly diagnosed glioblastomas was 5.0 months and the median overall survival for those with recurrent glioblastomas after PDT was 11.0 months (55).

A non-randomized controlled phase II study evaluated temoporfin mediated PDT in 26 patients with recurrent glioblastomas. Prior to enrollment, all patients had received standard surgical, chemo and radiation therapy. The PDT consisted of FGS (classified as a macroscopically total resection in 75.0% of cases) followed by the administration of intraoperative PDT. The median survival was 8.5 months, and the 2-years survival rate was 15.0%. The median survival rates for the PDT treated patients was significantly better than the survival in the control group (44).

### Efficacy of PDT for Glioblastoma

Despite sample size limitations and few randomized controlled studies of PDT, the data suggest potential beneficial effect of PDT for improving survival in glioblastoma patients when compared to standard therapy. The extent of PDT's practical application is hindered by the depth of light penetration into brain and tumor tissue with an estimated effective therapeutic spatial window for PDT limited to ~0.75–1.5 cm from the light source (16, 41, 61). At this time given the above limitations of light delivery and photosensitizer properties, aggressive tumor resection is necessary prior to application of PDT except in the case of very small tumors.

### Safety of PDT for the Treatment of Glioblastomas

Beyond complications associated with brain tumor resection, adverse events uniquely related to PDT include the systemic administration of a photosensitizer, the application of photostimulation and photochemical reactions. Each photosensitizer confers a slightly different safety profile. A risk common to all photosensitizers is retinal and cutaneous photosensitivity which occurs for several days in the case of 5-ALA to up to 6 weeks for temoporfin, during which time exposure to direct sunlight should be avoided (23).

A phase I–II study conducted by Kaye et al. that examined the efficacy of high-dose PDT using the photosensitizer HpD in 23 patients with malignant brain tumors found no evidence of increased cerebral edema nor any other adverse events, including hematological, hepatic and renal dysfunction (42). In another case series of 20 patients, side-effects of HpD administration included one patient with dermatotoxicity consisting of swelling of the head and hands after sun-exposure despite the application of sun blocking agents, which lasted for 1 week and three patients experienced meningeal symptoms (stiff neck, fever, headache) after direct injection of HpD into the tumors which lasted for 3 days (43). There was no mortality associated with PDT, however, three patients experienced symptomatic cerebral edema that responded to medical management. Another series reports a cerebral edema incidence of 0.04% cerebral edema after HpD PDT (58).

Reports of stereotactic iPDT suggest it is safe when applied to appropriately selected patients. One of the primary considerations is post-iPDT edema. In the experience at Kashiwaba Neurosurgical hospital with porfimer sodium mediated iPDT, cerebral edema was observed post-operatively in 46.0%, though the swelling was mild and did not require therapeutic intervention in 42.0% of cases (14).

Evaluation of 112 brain tumor patients treated with porfimer sodium mediated PDT yielded adverse events in 25.0% of cases (48). Among the adverse events were death (2.7%), post-operative hemorrhage (2.7%), neurological deficit (6.2%), deep venous thrombosis (3.6%), infection (3.6%), and light sensitivity reactions, such as hand burns, facial erythema, and facial pruritus 3.6% of patients. The majority of adverse events, however, were surgical and not directly related to photo-irradiation except for light sensitivity (48).

In a series of 365 PDT applications with 5-ALA and porfimer sodium in 150 brain tumor patients, adverse events occurred in 4.7% of patients (40). Deep venous thrombosis occurred in 2.0% of patients after administration of porfimer sodium, while no cases were observed after 5-ALA administration. Serious skin photosensitivity reactions developed in 1.3% of patients after non-adherence to light protection precautions. The photosensitivity reactions were considered avoidable had precautions been taken. Cerebral edema occurred after porfimer sodium mediated PDT in 1.3% of patients with recurrent tumors requiring intervention. In 0.7% of patients, there was rupture of the balloon diffuser used for photo-irradiation during PDT or cerebrospinal fluid leak (40).

In another series of 41 patients treated with porfimer sodium or temoporfin PDT, two patients with thalamic tumors died post-operatively due to significant post-treatment cerebral edema (59). Several adverse events were encountered during the trial. First, one scalp burn required plastic surgery with a cutaneous graft for repair. Other cutaneous toxicities were observed including blisters on the forearm and cutaneous erythema. A case of burn injury on the thumbnail from pulse oximeter was observed (59).

In another large case series of 100 patients with primary brain tumors who underwent HpD or porfimer sodium PDT, the authors report a mortality of 3.0% and combined serious morbidity-mortality rate of 8.0% (50). Of note, mean post-operative ICP is significantly higher after HpD or porfimer sodium mediated PDT compared to control patients (46).

Overall, porfimer sodium PDT has been associated with more adverse events than other photosensitizers, such as 5-ALA. For example, porfimer sodium is associated with increased risk of neurological deficits at a total photo-irradiation dose above 4,000 J (62). However, the apparent increased risk of adverse events with porfimer sodium mediated PDT compared to the use of other photosensitizers PDT may be due to the larger number of patients treated with porfimer sodium PDT.

There have been no systematic reviews of PDT safety. Overall, the common risks of PDT are retinal and cutaneous photosensitivity after administration of the photosensitizer. However, the risk of photosensitivity reactions are time limited and may be mitigated by avoidance of direct sun-light. The most serious safety risk of PDT is uncontrolled cerebral edema. The exact rate of cerebral edema after PDT is not known since it varies by photosensitizer as well as with the mode of delivery and intensity of photo-stimulation utilized during therapy.

## Emerging Technologies, Advancements In PDT For Glioblastomas

### PDT Mediated Immune Response

PDT has several unique properties that induce effective anti-tumor responses, such as apoptosis, autophagy, and necrosis as well as immunogenic cell death (ICD) (8). The unique modes of cell death evoked by PDT are thought to underlie a robust tumor-specific immune response which potentially leads to sustained immune mediated surveillance and suppression of neoplastic cell growth.

A mouse model cured of brain tumor by PDT provided the first evidence for induction of a tumor-specific immune response by resisting subsequent tumor cell re-challenge a tumor-specific manner while immunosuppressed mice did not resist the re-challenge (63). Several years later, the cellular mechanism of the anti-tumor immunity was further elucidated. Among the different modes of PDT induced cell death, ICD is a type of cell death whereby neoplastic cells expose and/or release of tumor antigens molecules known as damage associated molecular patterns (DAMPs) (64, 65) which activate both innate and adaptive immune responses (66). DAMPs are integral components of cells that are only exposed on the plasma membrane and/or released in response to injury, such as the oxidative damage caused by PDT. DAMPs play a key role in cell mediated immunity by causing the activation and stimulation of antigen processing/presentation by antigen presenting cells (APCs). The activation of APCs causes their migration and proliferation in local lymph nodes where the APCs then present the tumor antigens to CD8^+^ T cells (8). Activated CD8^+^ T cells actively surveil the body for neoplastic cells and induce apoptosis whenever tumor cells are encountered, thereby providing long-term tumor control. Therefore, ICD induced by PDT has the potential to stimulate immune activation and surveillance, contributing to long term tumor control is observed in pre-clinical models (63, 66–69).

Overall, the survival benefit of PDT trials for malignant gliomas has been modest, thus providing the rationale for adjuvant therapies, such as further augmentation of the immune response initiated by PDT. There have been few experimental studies exploring the effects of direct PDT mediated immune response on brain tumor growth and to our knowledge no clinical studies to date.

### Nanoparticle Photosensitizers

Nanoparticle technology provides several opportunities to improve upon the delivery, bio-availability, selectivity, and functionality of currently available photosensitizing molecules while reducing side-effects (70–72). Selective drug delivery using nanotechnology is an area of active research that may provide functional tumor cell type specific delivery capabilities as well as provide improved systemic pharmacokinetics of photosensitizer molecules (73–75). For example, nanoparticle-conjugated photosensitizers are in development to exploit tumor specific cell surface receptors which would deliver the photosensitizer directly to the tumor cell (76). The goal is to develop nanoparticles that are able to cross the BBB and selectively enter tumor cells. The availability of a photosensitizer capable of crossing the BBB and selectively entering tumor cells would broaden the spectrum of brain tumor targets to lower grade tumors for PDT as the requisite tumor mediated disruption of the BBB would be eliminated.

Upconverting nanoparticles, nanoparticles that convert multiple incident photons (generally in the infrared range) into an emitted photon (in the visible light range) of higher energy for PDT is an area of active research (77, 78). Another shortcoming of clinically available photosensitizers is the peak excitation wavelength necessary for activation requires wavelengths of light that poorly penetrate brain tissue. To address this limitation, nanoparticles are in development that are activated by deeper-penetrating near infrared light which causes the nanoparticles to release photons at the photosensitizer excitation wavelengths (78). The goal is to achieve a higher degree of tumor cell specificity (even in regions with intact BBB) while being able to apply PDT at greater distances from the light source and, therefore, over a larger tissue volume than can be achieved using current PDT techniques.

## Conclusion

Following on the heels of the recent FDA approval of 5-ALA for fluorescent guided glioblastoma resection, there is emerging interest in leveraging this agent toward administering photodynamic therapy (PDT) to the resection cavity. Review of the available literatures suggests that such PDT can be safely delivered to prevent local tumor recurrence. However, it is difficult to extrapolate the efficacy of the regimen given significant heterogeneity in study design, patient cohort, and PDT agents. This review provides data spanning over 25 years of technological sophistication of PDT, hence overlaps with extension in life expectancy and quality of life parameters conferred to glioblastoma patients by optimization of their multispecialistic care are difficult to evaluate. However, lack of clear efficacy of PDT in overall survival has limited the wide-spread adaption of this technology and implementation as a standard treatment of glioblastoma. Furthermore, technical limitations in light delivery and photosensitizer design have blunted the impact that this technology might have in the treatment of glioblastoma.

The immunological effects of PDT is of particular interest given recent studies demonstrating the importance of these processes in glioblastoma. Further studies of PDT in glioblastoma after stratification of pertinent molecular biomarkers, including isocitrate dehydrogenase mutation status and methyl-guanine-methyl transferase (MGMT) promoter methylation status is warranted. Incorporation of PDT into the current standard-of-care therapy should be explored in this context. Furthermore, exploration of next generation photosensitizer agents with increased specificity to glioblastoma is equally warranted.

## Author Contributions

All authors listed have made a substantial, direct and intellectual contribution to the work, and approved it for publication.

### Conflict of Interest

The authors declare that the research was conducted in the absence of any commercial or financial relationships that could be construed as a potential conflict of interest.
